# Internet Usage by Low-Literacy Adults Seeking Health Information: An Observational Analysis

**DOI:** 10.2196/jmir.6.3.e25

**Published:** 2004-09-03

**Authors:** Mehret S Birru, Valerie M Monaco, Lonelyss Charles, Hadiya Drew, Valerie Njie, Timothy Bierria, Ellen Detlefsen, Richard A Steinman

**Affiliations:** ^4^Bidwell Training CenterPittsburgh PAUSA; ^3^University of Pittsburgh School of Information SciencesPittsburgh PAUSA; ^2^Department of MedicineUniversity of PittsburghPittsburgh PAUSA; ^1^Department of Basic ResearchUniversity of Pittsburgh Cancer InstitutePittsburgh PAUSA

**Keywords:** Literacy, health, reading, Internet, health education, health promotion, socioeconomic factors

## Abstract

**Background:**

Adults with low literacy may encounter informational obstacles on the Internet when searching for health information, in part because most health Web sites require at least a high-school reading proficiency for optimal access.

**Objective:**

The purpose of this study was to 1) determine how low-literacy adults independently access and evaluate health information on the Internet, 2) identify challenges and areas of proficiency in the Internet-searching skills of low-literacy adults.

**Methods:**

Subjects (n=8) were enrolled in a reading assistance program at Bidwell Training Center in Pittsburgh, PA, and read at a 3rd to 8th grade level. Subjects conducted self-directed Internet searches for designated health topics while utilizing a think-aloud protocol. Subjects' keystrokes and comments were recorded using Camtasia Studio screen-capture software. The search terms used to find health information, the amount of time spent on each Web site, the number of Web sites accessed, the reading level of Web sites accessed, and the responses of subjects to questionnaires were assessed.

**Results:**

Subjects collectively answered 8 out of 24 questions correctly. Seven out of 8 subjects selected "sponsored sites"-paid Web advertisements-over search engine-generated links when answering health questions. On average, subjects accessed health Web sites written at or above a 10th grade reading level. Standard methodologies used for measuring health literacy and for promoting subjects to verbalize responses to Web-site form and content had limited utility in this population.

**Conclusion:**

This study demonstrates that Web health information requires a reading level that prohibits optimal access by some low-literacy adults. These results highlight the low-literacy adult population as a potential audience for Web health information, and indicate some areas of difficulty that these individuals face when using the Internet and health Web sites to find information on specific health topics.

## Introduction

Although a tremendous volume of educational health materials is disseminated in the United States, not all Americans find this information accessible or usable. In particular, adults with poor health and low functional literacy face great risks of poor health outcomes and preventable disease progression [1-4]. While many low-literacy adults could benefit from enhanced health knowledge, most current health education materials are written at a 10th grade or higher reading level [3]. Inability to access or understand health education materials inhibits important preventive or treatment measures, and may decrease the likelihood of identifying a symptom of disease. Low *health* literacy is also a barrier to enrollment in clinical trials [5,6] and minimizes adherence to instructions given by health professionals [7]. These obstacles are compounded by low income levels pervasive in the undereducated population [1], which can prevent individuals from pursuing regular primary care, paying health insurance premiums, or purchasing medications when prescribed. Collectively, these factors help to explain why low-literacy adults are twice as likely to be hospitalized as individuals with high functional literacy [8].

The expense of poor health and low functional literacy on the health system is estimated at $73 billion each year [9]. High cost estimates have encouraged many health-care providers to search for innovative ways to improve health literacy. The Internet has been embraced as an easy-to-use, convenient, and comprehensive clearinghouse for information on diseases, disorders, treatments, and preventions. Even when receiving physician care, between 40% and 54% of medical patients use the Internet to learn about treatment options and tobetter understand their medical conditions [10].

However, the low-literacy population has largely been excluded from the veritable boom of Internet health resources. The expense of Internet services and personal computers may be too high for this population. In addition, most text-based health information on the Internet is too advanced to be optimally effective for low-literacy populations. On average, Internet health-education materials are written at a 10th grade or higher reading level, and 100% of English-language health Web sites examined in a 2001 study required at least high school-level reading proficiency [11,12]. Another study concluded that of 1000 Web sites reviewed, only 10 had a level of writing and content accessible to low-literacy adults [13]. Kalichman et al suggest that individuals who read English below a 6th grade level are not likely to make effective use of the Internet [14]. Further, Zarcadoolas et al report that complex Web features, such as animated links, may be challenging for low-literacy adults to identify and utilize [15]. The 1992 National Adult Literacy Survey (NALS) revealed that more than 90 million Americans either read at a low-literacy level or are functionally illiterate [1]; the paucity of Internet health resources appropriate for these individuals perpetuates discrepancies in health outcomes between the educated and undereducated.

While no studies to date have determined how many low-literacy adults regularly use the Internet to find health information, the dearth of educational materials suitable for these individuals may impair optimal usage and navigation. One study has reported interventions that enabled low-income HIV-positive individuals to use the Internet and to critically evaluate information that they encountered [16]. Health-related Internet use has also been shown to enhance knowledge about HIV and to be correlated with active coping in a study of HIV-positive patients [17]. Although these studies focus on low-income status rather than low-literacy status, the established correlation between these two factors suggests that low-literacy adults may likewise benefit from augmented health education via the Internet.

We conducted an observational study of low-literacy adults to assess how they searched for Internet health information in as close to a natural setting as possible. Our investigative questions include the following: if low- to mid-level literacy adults are given access to the Internet, can they find basic health information that they can understand? Will their search strategies be effective in identifying information that they can use and comprehend? How do they rate current health Web sites in relation to their needs and interests? Will they be able to conduct successful self-directed searches? In our investigation, we also categorized navigational strategies used by low-literacy adults and the reading level of materials they accessed.

## Methods

We enrolled 13 adult literacy students (3rd to 8th grade reading levels) from Bidwell Training Center, a vocational school in Pittsburgh, Pennsylvania. The protocol used was approved by the University of Pittsburgh Institutional Review Board. Bidwell students are organized individually and/or in small groups for reading instruction; they meet together once a week for program announcements. The literacy program coordinator introduced the study to 20 students in this large-group setting. Thirteen interested students then self-selected into the study. All subjects participated in a computer skills workshop in May 2003, where they were presented with basic search and navigation strategies and learned how to use the Google search engine. We selected Google because it is a widely used search engine and has a "Did You Mean…" feature that corrects misspelled search terms. We anticipated that this might be a feature that low-literacy subjects would find particularly helpful. Among other topics, subjects were taught how to use the "Back" button and the "Forward" button, how to scroll down a page, how to identify links, and how to conduct basic searches. Each subject also filled out a brief questionnaire to give insight on their educational background, ethnicity, health insurance status, and previous experience with computers and the Internet. The questionnaire was written at a 3rd grade reading level (Flesch-Kincaid Reading Scale).

An investigator met individually with each of the participants within 3 weeks of the computer skills course for the observational portion of the study. Participants were 1) administered the REALM test (Rapid Estimate of Adult Literacy in Medicine) [18] to assess their health literacy level; 2) asked several questions to gauge their comfort level on the Internet and prior Internet experiences; and 3) taught how to "think aloud," or continually express their thoughts while using the computer. Investigators engaged each participant in several think-aloud examples in order to actively illustrate this process.

The investigator then asked the participant to use the Internet and Google search engine and think aloud while finding information on a subject of his or her choice. This preliminary question allowed participants to practice and review their Internet searching techniques. Participants were permitted to ask the investigator technical and navigation-related questions during this part of the study. These questions included, but were not limited to, whether to put spaces between words in search terms and how to initiate a search once a search term had been specified.

Participants were then asked to find answers on the Internet to 3 health-related questions developed by members of the research team. Participants were instructed to use the Google search engine so that their answers could be standardized. A committee consisting of a physician, a faculty member specializing in human-computer interactions, a community health educator, and an information sciences specialist compiled various answers to these questions that would qualify as accurate and complete. Subjects who were able to generate any of these answers during their online searches were considered to have answered the questions correctly; subjects who were not able to generate these answers were determined to have answered the question either incorrectly or incompletely. Examples of responses for each question that would have been considered correct are included in the *Results* section

The investigator read the 3 questions aloud and also provided them to the participant in written form (Arial font, 20 pt):

1. Think of a health question you are interested in for yourself or for someone you know. Find out information about this question on the Internet.

2. Imagine that someone you care about has lung cancer. This person would like to know about treatments for lung cancer. Can you find out the three main types of treatments using the Internet?

3. Imagine that you are at a doctor's office and you are told you have a disease called diabetes (sometimes called sugar). You are given a pill called Metformin to take for it. What does Metformin do?

Subjects identified answers to the investigator, who then asked them to articulate the answers in their own words. Participants who seemed frustrated or unreceptive, or who asked to move to a new question were directed to the next task. Participants were allowed to use any Web sites they felt would help them answer the questions. Participants also were not provided with dictionaries-our objective was to examine how they navigated the Internet without assistance from external sources. Subjects were given up to 15 minutes to complete each task, as measured by the investigator. To minimize anxiety, they were not informed of the time limitation. After the 15-minute period, investigators used a series of prompts to gradually guide subjects, if necessary, to the next task.

Next, investigators accessed the colon and rectum cancer Web page on the American Cancer Society (ACS) Web site [19]. Participants were asked to navigate through links on this page and find 2 ways to help prevent colon and rectum cancer. Investigators recorded the amount of time spent answering this question and the number of links participants clicked on to find the answers. After this task was completed, investigators asked the participants several subjective questions to qualify their experience on the Internet. Participants were then given $25 compensation, which ended their direct involvement in the study.

Investigators wrote notes on each participant's progress, and asked for participant feedback about the Internet both before and after searching the Internet. Investigators did not coach subjects on proper technical or navigational techniques after the initial practice question until subjects had completed their tasks. In 2 cases, investigators directed subjects to Google's "Did You Mean…" search term correction option in order to adjust for spelling mistakes; these subjects had repeatedly demonstrated very poor spelling proficiency before this intervention.

Camtasia Studio screen-capture software recorded individual keystrokes and think-aloud recordings. Questionnaires and think-aloud methods were used to ascertain the criteria used by participants in evaluating Internet health Web sites. Investigators also calculated the 1) literacy levels of Web sites accessed by the participants, 2) the amount of time spent on each Web site, 3) the number of questions answered thoroughly and correctly by each participant according to pre-determined standards, 4) the average number of sites used to answer each question, and 5) the number of participants who accessed sponsored sites, or paid advertisements appearing on the Google retrievals page, and how many used that information to answer questions.

## Results

Qualitative and quantitative results were analyzed in this study.

### Participants

In this study, the subject population was reduced from 13 to 8. Two participants were excluded because they did not attend the one-on-one searching session with the investigator. Two other participants were excluded because they were non-native English speakers who did not understand the tasks presented to them. One participant was later excluded because technical problems prohibited the retrieval of her computer searches.

The average age of our 8 remaining participants was 41.5 years. Five subjects were male and 3 were female. Seven identified themselves as African Americans and 1 self-identified as of Asian descent. The Asian participant was an English-as-a-second language (ESL) speaker with a university education from his native country. Seven of the 8 participants reported having health insurance. Seven of the 8 also had at least some high school or trade school education; 1 participant did not report educational experience on the intake questionnaire.

Of these subjects, 2 reported on the intake questionnaire that they had never previously used a computer or the Internet. Two reported that they had previously used a computer, but had not used the Internet. Subjects generally used computers with greater frequency than they used the Internet. Three participants reported on the questionnaire that they used the Internet 2 or more times a week; they later said verbalized that their main online interests were news, sports, cars, and/or entertainment information. The other 5 participants reported on the questionnaire that they used the Internet either occasionally or not at all. Usage reports from the intake questionnaires are provided in Table 1.

**Table 1:** Self-reported, written questionnaire responses about prior Internet and computer usage by subjects (n=8)

Subject	*Have you ever used a computer? If so, how often?*	*Where do you use computers?*	*Do you use the Internet? If so, how often?*	*Where do you use the Internet?*
1	No	"No where" [sic]	No	"I've never used the Internet"
2	Less than once a month	"When I was in jail"	No	(N/A)
3	2 or more times a week	"At school, Bidwell Training Center in Ms. Cooper's class."	No	"At the Carnegie Library in Beechview where I live"
4	No	"No"	No	"No"
5	2 or more times a week	"To type"	Yes; Less than once a month	"In school"
6	No; 2 or more times a week	"At home"	Once a week	"At home"
7	2 or more times a week	"Home"	2 or more times a week (at home)	"Home"
8	Once a week	"Different location"	2 or more times a week	"Different location"

As seen in Table 1, the self reports of prior Web and computer experiences are unclear in several cases. Subject 3 reported no prior Internet usage in one part of the questionnaire, but reported in a subsequent answer Web usage at a local public library. In addition, as Table 1 indicates, subject 8 reported more frequent usage of the Internet than of computers; subject 6 (ESL student) first indicated no prior computer usage, then later reported on the questionnaire computer usage of twice a week. Because there were seemingly divergent perceptions of what constitutes a computer or Internet experience, perceived computer/Web adeptness cannot be correlated with our participants' experience using this technology. Therefore, while this study will indicate differences in results between the 3 people with frequent Internet experiences (defined in this study as usage of at least once a week) and the 5 individuals without, the study will not attempt to conclude whether the skill level of subjects in the study correlated with the sustainability of their prior computer and Web experiences.

### Search Engine Usage

Participants reviewed their navigational skills during their preliminary question, where they were encouraged to look for information on any subject that interested them. They used Google to search for a variety of topics, ranging from entertainment to health-related information. Participants occasionally searched for information on more than one topic.

Participants used the search items listed in Table 2 in order to answer the preliminary question and questions 1 to 3. Semicolons between words or phrases separate multiple search terms used by a subject to answer a question. The subjects are listed in Table 2 in the same order (ie, 1, 2, 3…) as they appeared in Table 1.

**Table 2:** Search terms used by subjects to answer preliminary questions and questions 1 to 3 (n=8)

Subject	Preliminary	Question 1	Question 2	Question 3
1	lena horn†	Lung cancer	Lung cancer	Metformin
2	health care;health care mental	Sports and health	health care about lung cancer	A pill called metformin
3	*(no clear search topic)*	Herpes	Cancer	Metformin
4	Wwwsoulfood; wwwsoulfoodcom; soulfood	AIDS	lung caner†	Diabetes
5	Will Smith; sipers†; spiders	High blood	Lung cancer	Metformin
6**	Bi;;;dwell training center†	Health	health lung caner†	Health diabetes
7	sonny Rollins	Tuberculosis	Treatments for lung cancer	Metformin
8	Babyface recording artist	Pain	Cancer	Pdr*

^*^ Physicians' Desk Reference

^**^ English-as-a Second Language subject

^†^ misspellings for: "lena horne," "bidwell training center," "lung cancer," and "spiders"; the Google correction option was used in two instances when the subject was prompted by investigators to amend search terms.

Questions 1 to 3 were given to our participants in writing, as well as orally; this may have affected their selection of search terms. For question 2, one participant wrote "treatments for lung cancer" in the search term box, a phrase that is written explicitly in that question. Another participant was similarly prompted by the wording of question 3 to write "a pill called metformin" as his search term.

Individuals who used the Internet at least once a week are labeled in Table 1 and subsequent tables as subjects 6 to 8. Search terms generated by these frequent Internet users did not differ greatly from search terms generated by individuals who had little Internet experience. The one exception was subject 8, who attempted to answer question 3 by using the online *Physicians' Desk Reference*, a site about which she had once heard good reviews.

In general, this group found generating original search terms to be somewhat challenging. Many did not initially remember whether to put spaces between the words in search terms. Even a subject who reported using the Internet once a week hesitated when writing the search term for question 1, finally stating, "*Yeah, you do have to space [between words]… I had to remember if you had to space.*" With one exception, participants were able to correct their terms by inserting the proper spaces.

Spelling of search terms was generally a problem for only 2 participants, one of whom (subject 7) spoke English as a second language. Subjects tended to self-correct for spelling in the search term box before pressing the "Google Search" button or Enter key. Several participants also had difficulty understanding what type of terms to put in. When conducting a preliminary search for information on the television show, *Soul Food*, one participant typed into the search term box, "wwwsoulfood." When this retrieved no results, the subject looked at the URL for guidance and then typed "wwwsoulfoodcom" into the Google search term box. This again did not yield any results. The participant next entered "soulfood" into the search term box. The investigator finally directed the subject to Google's "Did You Mean…" option so that the subject could answer the question. However, this participant had continued difficulties generating correct search terms; later in the study, he used "lungcaner" as a search term to find information about lung cancer.

Nearly all participants retained skills such as scrolling and clicking on links from the computer workshop or previous Internet experiences. They also learned other navigational strategies through repetition and practice. For example, one participant who was conducting a preliminary search for information about Will Smith looked at the Google retrievals and stated, "*So it [search engine] must go to other Smiths* ... *I wonder if I was supposed to put in 'Will Smith the actor'?*" Quickly, the subject had learned that increasing the specificity of search terms generally improves the specificity of results.

Six of the 8 participants did not venture past page 1 of the Google retrievals. One participant was surprised by the number of search results, saying, "*You find a lot of stuff on this thing [the Internet].*" Another participant explained why she stayed on page 1: "*Oh boy, I've got a lot to choose from. I don't want to go to the other ten [pages of retrievals] because it might give me other information I don't really need* ... *the first page gives me just enough of what I need to know.*" This participant had deduced that first-page retrievals typically have the most relevant sites to the particular search term used. Later, this subject stated, "*I didn't answer the questions, but I looked up the information, and it [Internet] gave me what it wanted me to have.*" This statement implies that the subject believed that the Internet was more in control of the searching than the subject, revealing a possible belief that the search engine and search terms selected are not the primary determinants of what type of information is retrieved.

### Sites Accessed

#### Ability to Answer Questions

In question 1, participants were asked to use information on the Internet to find the answer to a health-related query of their choice. Most participants identified only a subject area, and did not clearly articulate a specific question despite verbal prompting by the investigators. Several participants initially stated a topic, but changed it as they retrieved unrelated material that they found more interesting. While recordings from the think-alouds would have been helpful in designating the search topics, we found that despite investigators' prompts and encouragement, subjects were very reluctant to verbally report their real-time experiences navigating through the Web. As one subject stated, "*Shucks, I can't think aloud.*" It is therefore difficult to gauge whether participants were able to find adequate information for which they searched, especially during the unstructured searching period required to answer the first question.

Question 2 required participants to locate the 3 main types of lung cancer treatments (acceptable answers: chemotherapy, surgery, radiation). This question models the navigation of a typical Internet health-information seeker who searches for disease-related information. Of all 8 participants, only subject 5 was able to answer this question accurately and completely. Subject 3 verbalized one viable option-chemotherapy-based on information accessed online. The remaining participants either did not answer the question or identified an alternative medicine as one of the principal types of lung cancer treatments available.

Question 3 required participants to find out the role of metformin, or Glucophage, in diabetes treatment (one acceptable answer: metformin lowers sugar in the blood). This question models a doctor-patient interaction in which a patient who is prescribed an unfamiliar medication independently searches for information about its effects. Six of 8 participants were unable to find information on the Internet to answer the question. The 2 participants, subjects 3 and 7, who found the information, read directly from text on the site and did not articulate the information in their own words.

Surprisingly, subjects who reported sustained prior Internet experience in the questionnaire were no more successful at answering questions than subjects with little Internet experience. This could have been a result of the generalized search terms that they used to look for answers. Prior Internet experience does not seem to lead to satisfactory search/navigation skills for members of this group in searching for health information.

#### Information Accessed

Sites used by subjects 3, 5, and 7 to successfully answer questions 2 and 3 were written at a 12th grade reading level (Flesch-Kincaid). It is noteworthy that these subjects were able to identify the answer in the text and read it aloud. In 2 out of 3 cases, they were unable to express these answers in their own words, which suggests a minimal comprehension of the material accessed.

Seven of the 8 participants accessed sponsored site information while attempting to answer questions. Businesses pay a service fee to Google to have their site names appear as sponsored sites when triggered by a particular search term or keyword. Sponsored sites are outlined in color and/or appear in boxes on the right side and heading of the Google retrievals page. In general, alternative treatments and commercial therapies and medications appear under this listing; many of these sites may contain information that is uncorroborated by legitimate scientific sources.

Five participants used information provided by the sponsored sites to answer questions. Two out of 3 of the subjects who used the Internet at least once a week also used this information to answer questions. Half of the participants searching for lung cancer cures arrived at the same site: an Asian dietary supplement site claiming to cure cancer by removing free radicals from the body [20]. Another popular sponsored site promoted a radio frequency technique to hinder cancer progression [21]. The titles of these sites as they appeared in the sponsored sites submenu were: "New Cancer Treatment" and "Cancer Treatment." The Flesch-Kincaid formula indicated that the information on both sites was written at a 12th grade or higher reading level. Information on sponsored sites, therefore, was not necessarily any easier to read or interpret than information on non-sponsored sites accessed by subjects in this study.

#### General Site Profiles

Observational logs and records on the Camtasia software show little correlation between our subjects' ability to identify answers and the amount of text on a page; analysis using the Camtasia software also showed little conclusive difference in the amount of time that the subjects spent on each site despite variances in the amount of text on the pages accessed. Therefore, subjects did not seem to prefer or navigate towards Web pages/sites with less text.

Participants, on average, used between 1 and 2 Web sites to answer questions 1 to 3. Table 3 records the number of links from the Google retrievals page that were selected by subjects. The results for subjects 1 to 5-the participants with minimal prior Internet experience-are also presented separately from the results for participants with sustained prior Internet experience (subjects 6 to 8).

The Flesch-Kincaid reading scale used in this study scores text at a 1st to 12th grade reading level. Given this scale, sites ranked at the 12th grade level require *at least* that level of reading ability. That is, material scored at a 12th grade level may actually be written at a college level. In our study, the average site accessed required at least a 10th grade reading level.

**Table 3:** Average number of links used to answer questions

	Avg. Number of Links Used (Average Total)	Avg. Number of Links Used (Subjects 1-5)	Avg. Number of Links Used (Subjects 6-8)
Preliminary	1.875	2.4	1.0
Question 1	1.14	1.2	1.67
Question 2	1.82	1.8	2
Question 3	1.5	1.6	1.33
AVG.	1.58	1.75	1.5

**Table 4:** Average (rounded) reading level of sites accessed

	Avg. Reading Level of Sites Accessed	Avg. Reading Level of Sites Accessed (Subjects 1-5)	Avg. Reading Level of Sites Accessed (Subjects 6-8)
Preliminary	10.50	10.7	10.0
Question 1	10.50	9.4	11.2
Question 2	11.1	11.3	11.0
Question 3	11.8	11.8	11.9
AVG.	11.0	10.8	11.0

**Table 5:** Average time spent on sites

	Avg. Total Time Spent Per Site (min)	Avg. Total Time Spent Per Site (Subjects 1-5)	Avg. Total Time Spent Per Site (Subjects 6-8)
Preliminary	7.2	8.7	4.7
Question 1	10.3	10.6	9.8
Question 2	8.7	8.7	8.7
Question 3	6.6	8.3	5.8
AVG.	8.2	9.1	7.25

Participants spent an overall average of 8.2 minutes on individual sites. All participants voluntarily finished answering questions 1 to 3 before the 15-minute time limit was reached.

After completion of these first 3 questions, subjects were directed to a specific site; question 4 was posed about information directly linked to that site. We chose to use the ACS colon and rectum cancer Web page site, which contains links to a variety of prevention resources written at 6.3-12.0 grade levels (Flesch-Kincaid Reading Scale). The page to which we directed subjects consists of a listing of links to defined topic areas, one of which was closely related in wording to question 4. On the ACS site, 5 out of 8 people were able to answer question 4 correctly. Three of the 5 reported prior Internet experience; 2 reported none. These subjects used 3.8 links on average to answer the question. The 3 subjects who did not access the material used 6.5 sites on average before they were either stopped by the investigator or quit voluntarily. Two of these subjects had never used the Internet prior to enrollment in the study.

#### Attitudes and Self-reporting

While most participants were unable to answer all of the questions asked, 7 out of 8 reported feeling very comfortable or comfortable with their Internet searching experience. The eighth participant felt moderately comfortable. Also, 5 out of 8 found it at least moderately easy to find readable and understandable information on the Internet. Two of the remaining participants found it very difficult to find readable information, and one participant reported that finding understandable information is easy if the Web user has strong reading skills.

Despite their dependence on sponsored sites and alternative Web sites to answer questions, 7 out of 8 subjects reported that they found it very easy to locate trustworthy information on the Internet. The eighth subject noted that it is moderately easy to find information that is trustworthy on the Internet. However, one subject said, "*I believe that on the Internet, you have your shysters* ... *just like anything.*" 

Subjects felt positive about continuing their online experiences, and all expressed some enthusiasm about improving their skills. One participant stated, "*I'm getting a computer* ... *it can help your typing skills.*" Another subject said, "*The computer is real interesting. I'm a see if I can get one so I can learn [how to use it].*" After the study was completed, many participants asked investigators to continue teaching them Internet skills or to continue helping them locate Internet resources on a variety of subjects.

## Discussion

This observational study is the first to examine Internet use by low-literacy adults seeking health information [11]. Irrespective of prior experience using the Internet and/or computers, low-literacy adults participating in our study did not use optimal search terms to answer questions, encountered difficulties finding health information at the appropriate reading level, and were unable to successfully interpret Internet health information as it was presented. While basic navigational skills (eg, using the "Back" button) were easily retained, areas that required reading and comprehension were problematic for most subjects-evidenced by their inability to answer questions and comments made during their think-alouds. Therefore, the literacy level needed to read health information on the Internet does appear to inhibit information-seeking efforts of low-literacy adults.

Searching strategies were sub-optimal in several respects. First, the search terms used by subjects were predominately non-specific (Table 2). Although we anticipated that subjects who used the Internet more often would generate more specific search terms than did their peers, we did not observe this in the study.

### Difficulty Generating Search Terms

Without guidance, subjects had difficulty generating original search terms that would yield specific results. A recent study reveals that adolescents used similarly general search terms when searching the Internet for health information [22]; this corroborates results from another study, which found that among subjects with an average of 33 months of Internet experience, self-selected search terms to find health information were unexpectedly general [23]. These observations highlight search terms as a potential barrier to specific, targeted Internet health information for different types of Internet users with varying levels of Web expertise. A categorizing search engine might be particularly effective for use by these groups; it minimizes the need for individuals to both create a specific search term and independently read and assess all retrievals. A sample search to answer question 2 was conducted using the Vivisimo search engine [24]. The search term "lung cancer" yielded a series of folders about lung cancer separated by subject matter; one folder specifically focused on lung cancer treatments. Individuals clicking on that option could access all sites on lung cancer treatments retrieved by the engine, circumventing the need to sift through thousands of retrievals to locate treatment-focused sites. A future study could monitor the ease with which low-literacy individuals could conduct self-directed searches using an automatically sorting search engine.

### Reluctance to Use Links

Search strategies observed in this study were also sub-optimal because most subjects exhibited some unwillingness to click on links to Web sites on the Google retrievals page. On average, subjects clicked on one to two links to answer questions. Even when the subjects did not appropriately answer questions or only partially answered questions, most seemed reluctant to click on additional links on the Google retrievals page, and 7 of 8 did not go to subsequent retrievals pages. These results did not seem to correlate with prior Internet experience. Subjects also rarely re-typed search terms in order to access more relevant retrievals. These results differ from those of a previous observational Internet study, whose participants preferred to choose links from page-one retrievals and then re-type original search terms if they were unable to find appropriate information [23]. As stated earlier, our subjects had such difficulty generating original search terms, figuring out appropriate spelling, and determining whether to place spaces between words in search terms, it is conceivable that this is why they avoided this strategy.

Another reason why subjects' generation of search terms and selection of links were so limited may have been because the subjects were not interested in the health materials or the questions. Subjects may have also found the Google retrievals page confusing and intimidating. While the think-alouds are inconclusive about which of these factors contributed most to the weak search strategies observed, the post-session questionnaire reveals that the majority of participants reported that it was easy to search the Internet. Future research may help to illuminate the factors that contribute to the inconsistencies between subjects' perceived unwillingness to explore the Internet's health resources and their positive feedback about navigating through these resources.

### High Literacy Levels of Health Web Sites

The *health* sites participants accessed to answer questions 1 to 3 had, on average, an 11th grade reading level (Flesch-Kincaid Reading Scale), which was consistent with the findings of previous studies [3,25]. Clearly, all of our subjects experienced difficulties using these sites to answer questions. The literacy level of the materials that the subjects did access may have limited their ability to read and understand materials as presented to them, and may have also impaired their ability to select the appropriate links for finding information. However, a majority of subjects were able to find specific information on the ACS Web site. As one subject reported about the site, "*This is a real good one 'cause it breaks it right down for you*." This Web page consisted of a series of links: general links on the left and right sides of the page and links to colorectal cancer in the center. Subjects who were unable to answer the questions seemed to find the lists of links on the page confusing, and picked links that took them to unrelated pages on the ACS site rather than to specific pages containing colon and rectum cancer information. While the selection of only 1 link on the colon and rectum cancer Web page was necessary in order to answer the question, these subjects on average picked more than 6 separate links before quitting. Therefore, layout of health Web sites evidently affects the ability of low-literacy adults to find pertinent health information.

Despite the navigational difficulties observed on the ACS Web page, the ability of 5 subjects to correctly answer question 4 probably resulted from the fact that the information needed to answer question 4 was written at an 8th grade reading level-significantly lower than the11th grade reading level required on average to read information retrieved in the first 3 searches. This suggests that low-literacy individuals can identify and utilize easier-to-read materials on Web sites. The Internet may indeed be a useful health resource to this population if materials are written at an appropriate reading level. Considering the navigational struggles of our subjects, the actual process of locating low-literacy sites on the Web may prove a more daunting challenge to this population.

### Difficulty Measuring Participants' Comprehension of Information

While most were able to competently navigate through lower literacy materials, subjects' comprehension of Internet health information was difficult to measure in our study. Some participants found correct answers and read them to the investigators directly from the Web text, but none were able to articulate the answer in their own words when prompted. In their analysis of the1992 National Adult Literacy Survey (NALS) results, Kirsch et al reported that low-literacy adults may successfully perform simple comprehension exercises such as locating a single piece of information from text, but often find it more difficult to integrate and synthesize that information [1]. Furthermore, subjects in our study may have been able to use cues from sentence structure to locate an answer, and then relied on their pronunciation skills in order to read the answer as written. However, their ability to identify relevant health information within text is not necessarily a measure of their ability to comprehend that information.

In addition, several subjects seemed to compensate for their low literacy skills by using external information resources. One subject who examined a Web site on mental health law (12th grade level) expressed great enthusiasm about a particular topic that he said was presented on the site. A perusal of the site after the session showed that this topic was not addressed on any of the pages he had accessed. This participant may have compensated for his struggles in reading the site by citing facts with which he was personally familiar. Another subject used a similar approach when accessing a lung cancer site. When asked about the type of information he was reading, the subject responded that the page focused on smoking cessation. However, there were no smoking-related topics on the pages examined by the subject. The subject was able to correlate lung cancer with smoking, and may have relied on this information in order to answer the investigator's query. Overall, some subjects may have been able to rely less on actual comprehension skills and more on background knowledge in order to infer answers.

Positive Web-site and performance feedback reported by most of the participants could have also been fueled by a desire to compensate for reading and comprehension difficulties. Participants were aware that the majority of the investigators were affiliated with a local hospital system; some may have felt compelled to answer positively about Internet health information because they were reporting to health-care professionals. Additionally, the participants may have been unwilling or ashamed to admit that they had difficulty understanding the information on the Internet. Individuals with low literacy tend to be embarrassed by their reading inadequacies [26]. Participants may have felt compelled to report more positively about their Internet experiences in order to de-emphasize their difficulties navigating the Web. These considerations might begin to explain that while most participants struggled when using the Internet, most 1) felt they did a good job searching for information, and 2) found information on the Internet readable and understandable. Collectively, then, poor comprehension of health information on the Internet coupled with a desire to compensate for self-perceived inadequacies in reading may have negatively affected the ability of our subjects to objectively evaluate Web sites. In this study, these factors may also have diminished the accuracy of their think-alouds and feedback in relation to their actual Internet experiences.

### Inaccurate Self-assessment

An alternative reason why subjects reported positive experiences on the Internet could be that subjects were unaware of the magnitude of their Internet searching difficulties. A study by Moon et al indicates that 70% of subjects told investigators that they read "really well," while in actuality, their mean REALM scores reflected a 7th to 8th grade reading level [27]. This suggests that individuals may actually overestimate their reading ability in relation to standard educational parameters; it may also relate to a similarly heightened perception of Internet competence. Furthermore, because the majority of our subjects had minimal Internet experience, they may not have been able to objectively gauge the limitations of their Internet skills in relation to the skills of more advanced users. While the investigators were able to categorize their searching as sub-optimal, our participants could have considered their searching strategies to be adequate, if not standard.

### Preference for Sponsored Sites

Subjects' reliance on sponsored-site information to answer questions, regardless of the high literacy level required to read those sites, suggests that other factors promote the selective advantage of sponsored sites over non-sponsored sites. In fact, the design of sponsored sites on the Google retrieval page follows many of the guidelines for creating optimal layouts for health information targeted to low-literacy adults [28]. First, the sponsored sites are organized by topic, and are also segmented in colored boxes that stand out from the rest of the Google retrievals. They do not contain the "teaser information" and keywords associated with normal Google links, and minimize the amount of text used. Most are easier to read than the normal Google links, are automatically categorized by subject, and are visually stimulating. In addition, despite misspellings of search terms, sponsored sites are often applicable to the intended subject. For example, a search of "lung caner" instead of "lung cancer" yields sponsored sites on lung cancer, though most of the non-sponsored Google retrievals are irrelevant. When individuals misspell search terms, which the low-literacy subjects in our study did fairly commonly, they might easily gravitate to sponsored-site information to answer their health questions.

Of concern is that subjects did not seem to differentiate between the information on the sponsored sites and information on non-sponsored sites. Subjects used these sites interchangeably to answer questions. One study suggests that critical interpretation of Web sites is based on the Internet acumen and interests of the information-seeker; if coupling the *motivation* to find a topic and the *ability* to do so successfully, the information-seeker will be well-equipped to evaluate Web sites objectively and perceptively [29]. This approach offers 3 possible explanations for our results. First, our questions may have been of little interest to our subjects; this may have diminished their motivation in answering questions and affected impacted their critical analysis of sites. Second, many of our subjects had little sustained exposure to various Web sites before the study. Those subjects in particular may not have been able to critically compare Web sites as readily as individuals who had previously seen both good and bad Web sites and developed their own rating system. In this context, most health information on the Internet may have seemed trustworthy and interchangeable to some of the subjects. Third, the searching problems observed even among those subjects with previous Internet experience underscore the fact that none of these subjects reported that their prior Web usage included searches for health information. While these subjects had successfully found items of personal interest in previous Web searches, they were unable to navigate to health materials that were any more accurate or easy-to-read than those found by the rest of the subjects. Therefore, health searches may present unique challenges to a low-literacy population that counter the ability to find accurate, trustworthy health information. This may result from the high literacy level required for reading health information and health Web sites in addition to the complexity of health terminology.

### Limitations of Methodology

Standard methodologies used in this study to determine health literacy and to generate continual feedback were sub-optimal. First, REALM test results were inconclusive. Subjects were placed into the literacy program at Bidwell Training Center after taking the national Tests for Adult Basic Education (TABE). However, in our study, these subjects tested significantly higher on the REALM than expected for individuals with the reading levels indicated by their TABE scores as reported by Bidwell Training Center (3rd to 8th grade reading skills). Subjects may have strong phonetic skills that help compensate for poor word recognition and comprehension. This observation is supported in a study by Wilson et al [30], which similarly noted that lower literacy participants who used the REALM tested at several grade levels above their actual reading level. The REALM may not be an optimal tool for accurately determining the health literacy of low-literacy adults.

Whereas complete think-alouds could have helped us better understand subjects' navigational priorities and comprehension levels, the protocols we used in this study were ineffective at prompting verbalization. None of the participants consistently articulated their step-by-step navigational process at all points during their searching session. Investigators continually prompted the subjects through the exercise, but were unable to stimulate free-thinking, consistent, and self-motivated think-alouds. One potential explanation originates from the observation that our study population was not uniformly familiar with the Internet. Therefore, some subjects may have felt overly challenged by simultaneously learning how to use the Internet and verbalizing their navigational strategies. According to previous studies [31], these subjects were probably in an "acquisition role." Such studies disclosed that a learner who is new to a certain task focuses primarily on acclimatization, and finds it overwhelming to concurrently think aloud. Since traditional think-aloud protocols may be ineffective for this group, an interactive protocol may be of assistance for future studies. In such a protocol, subjects would directly be asked about specific site features, and asked to rate and make comparisons between health sites. This may highlight precise preferences the subjects might have for Web-site information, content, design, and presentation, and may result in a more cohesive rating system.

Overall, however, our subjects were very enthusiastic about learning how to use the Internet, and all indicated an interest in improving their skills for future use. In this study and other studies [13,15], members of the low-literacy population have expressed excitement about using the Internet. In order for the Internet to further empower these individuals to make informed health decisions, the development of easy to read *and* easy to comprehend health materials is imperative. If Google's sponsored sites are usedas a guide, low-literacy adults prefer information that is aesthetically pleasing, has minimal text, and is organized by subject matter. Search engines that are able to consolidate these features for searches will probably be of greater use to this population. However, low-literacy adults must improve their navigation and searching skills to efficiently locate low-literacy materials on the Internet. With sufficient practice, they are likely to develop the skills to use the Internet to find specific health information, and learn to critically evaluate the information they access.

### Indications for Future Research

One caveat to the present study is that our sample size precluded the analysis of factors besides low literacy that could influence the results we observed. We believe, however, that our findings with this sample group in an observational study were representative of the way low-literacy adults interact with the Internet. It will be important to validate and analyze in a larger study the appeal of sponsored sites (as opposed to other retrieved links) to low-literacy adults. It will also be worthwhile to determine the relative importance of limited literacy in comparison to socioeconomic and cultural factors in effective use of the Internet by this population. Future work will identify the exact components of sites that engage and promote learning by low-literacy adults. Greater understanding of these factors will hasten the day when the Internet becomes an effective vehicle for optimizing the health knowledge and acumen for those at high risk of poor health outcomes.

## Abbreviations

ACS: American Cancer Society
NALS: National Adult Literacy Survey
REALM: Rapid Estimate of Adult Literacy in Medicine
TABE: Tests for Adult Basic Education
